# Effect of Transitional Metals (Mn and Ni) Substitution in LiCoPO_4_ Olivines

**DOI:** 10.3390/molecules25030601

**Published:** 2020-01-30

**Authors:** Oriele Palumbo, Jessica Manzi, Daniele Meggiolaro, Francesco M. Vitucci, Francesco Trequattrini, Mariangela Curcio, Annalisa Paolone, Sergio Brutti

**Affiliations:** 1 CNR-ISC, U.O.S. La Sapienza, Piazzale A. Moro 5, 00185 Rome, Italy; oriele.palumbo@roma1.infn.it (O.P.); manzi.jess@gmail.com (J.M.); Francesco.m.vitucci@gmail.com (F.M.V.); francesco.trequattrini@roma1.infn.it (F.T.); annalisa.paolone@roma1.infn.it (A.P.); 2 Computational Laboratory for Hybrid/Organic Photovoltaics (CLHYO) Istituto CNR di Scienze e Tecnologie Chimiche “Giulio Natta”(CNR-SCITEC), Via Elce di Sotto 8, 06123 Perugia, Italy; daniele.meggiolaro@iit.it; 3 Department of Physics, University of Rome ‘‘La Sapienza’’, Piazzale A. Moro 5, 00185 Rome, Italy; 4 Department of Sciences, University of Basilicata, V.le dell’Ateneo Lucano 10, 85100 Potenza, Italy; mariangela.curcio@unibas.it; 5 Department of Chemistry, University of Rome ‘‘La Sapienza’’, Piazzale Aldo Moro 5, 00185 Rome, Italy

**Keywords:** cathode materials, olivine, X-ray diffraction, X-ray absorption

## Abstract

Transition metal substitution is a key strategy to optimize the functional properties of advanced crystalline materials used as positive electrodes in secondary lithium batteries (LIBs). Here we investigate the structural alterations in the olivine lattice of Mn and Ni substituted LiCoPO_4_ phase and the impact on performance in LIBs. X-ray diffraction (XRD) and extended X-ray absorption experiments have been carried out in order to highlight the structural alterations induced by partial substitution of cobalt by manganese and nickel. XRD analysis suggests that substitution induces an expansion of the lattices and an increase of the antisite disorder between lithium and transition metal ions in the structure. XAS data highlight negligible electronic disorder but a relevant modulation in the local coordination around the different metal ions. Moreover, galvanostatic tests showed poor reversibility of the redox reaction compared to the pure LCP sample, and this failure is discussed in detail in view of the observed remarkable structural changes.

## 1. Introduction

**Figure 1:**
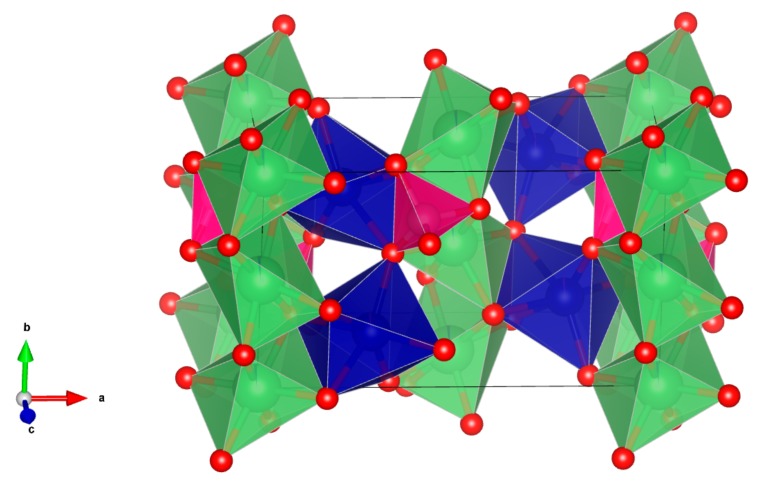
Of the octahedral unit cell of the olivine lattice (green octahedra: LiO_6_ units, blue octahedra: TMO_6_ octahedra, pink tetrahedra: PO_4_ units, TM = transition metals). Red full circles are oxygen atoms, green full circles are lithium atoms, blu full circles are TM atoms.

The improvement of lithium-ion batteries technology requires the development of alternative electrodes and electrolytes materials, able to provide better performances in terms of energy densities, cycle life, safety, and sustainability. Among various positive electrode materials, olivine compounds such as LiCoPO_4_ (LCP) or LiFePO_4_ (LFP) have been exploited, with the aim to improve safety with respect to the use of the LiMn_1.5_Ni_0.5_O_4_ spinel or of carbonaceous cathodes. LFP and all the related fluorophosphates crystallize in an olivine-type lattice (see Figure 1) where transition metal and lithium XO_6_ octahedra share corners with PO_4_ tetrahedra and lithium ions are aligned along the [010] axis. In particular, LCP is considered a promising cathode material for high energy Li-ion cells due to its very high working potential (>4.7 V vs. Li) [1], but, compared to the isostructural and commercially exploited LFP, LCP cathodes suffer poor reversibility in the first electrochemical de-insertion/insertion [2], a remarkable capacity fading on cycling [3,4], and a spontaneous self-discharge once fully charged [5,6]. Moreover, the LCP operates at the limit of the anodic stability window of any carbonate-based liquid electrolytes thus opening the door to remarkable degradation chemistry over the cathode surface upon cycling [6]. In order to mitigate these drawbacks, a partial substitution of the Co^2+^ has been suggested [1].

Various synthesis of LCP-based olivines with partial substitution of cobalt with iron (LCfP) has been proposed [1,7,8,9,10,11,12,13], obtaining a beneficial effect on the long-term cycling stability [7,8,9]. Despite large efforts, the interplay between the modification induced by substitution on the lattice structure and the improvement of the transport properties and of the reversibility of lithium insertion and de-insertion in the olivine lattice is only partial. Recently, improved and stable performances in lithium cells have been obtained using an LCfP material prepared by solvothermal synthesis followed by high-temperature annealing [3]. The analysis of the short- and long-range structural alterations induced by iron-substitution and high-temperature annealing showed a close relationship between the local coordination around the transition metals with the redox activity in lithium cells as well as with the ionic transport properties [14]. In particular, iron substitution followed by high-temperature annealing is found to alter the concentration of anti-site defects, of the natural concentration of lithium vacancies, of the size of lithium diffusion channels along the [010] direction as well as their local distortion [14], leading to an enhancement of the lithium diffusion coefficient and to an increase of electrochemical lithium extraction/insertion reversibility in lithium cells.

Going beyond iron, other similar first-raw transition metals have been considered as possible substituents in the olivine LiCoPO_4_ lattice. In particular, the substitution of Co by Ni or Mn has been previously studied computationally [15,16] and experimentally [17,18,19,20]. However, as far as we know, all the available reports miss a detailed analysis of the structural alterations of these substitutions, both in the short and long-range. Therefore, only a partial rationalization of the impact of Mn and Ni substitutions on the electrochemical properties is available, in contrast with the wide analysis already reported fro iron substitution [3,7,8,9,10,11,12,14].

In the present paper, we investigate the structure of LCP based cathode materials with partial substitution of the Co^2+^ with Mn^2+^(LCmP) and Ni^2+^(LCnP) ions. In order to ascertain the effect of high-temperature annealing, we compared these as-prepared materials with their analogous exposed to thermal treatment under Argon atmosphere (LCmP@Ar and LCnP@Ar). Considering the beneficial effect obtained by iron substitution on the long-term performances of LCP and its strong relation with the local coordination of the transition metal and the structural modifications induced by the substitution, in order to further ascertain this relation we selected these two elements (Ni and Co) among transition metals keeping iron as a benchmark since they present Shannon radii either lower (0.690 Å for Ni^2+^) than that one of Co^2+^ (0.745 Å), such as iron (0.645 Å for Fe^3+^), or higher (0.830 Å for Mn^2+^).

## 2. Results

### 2.1. X-Ray Diffraction

**Figure 2:**
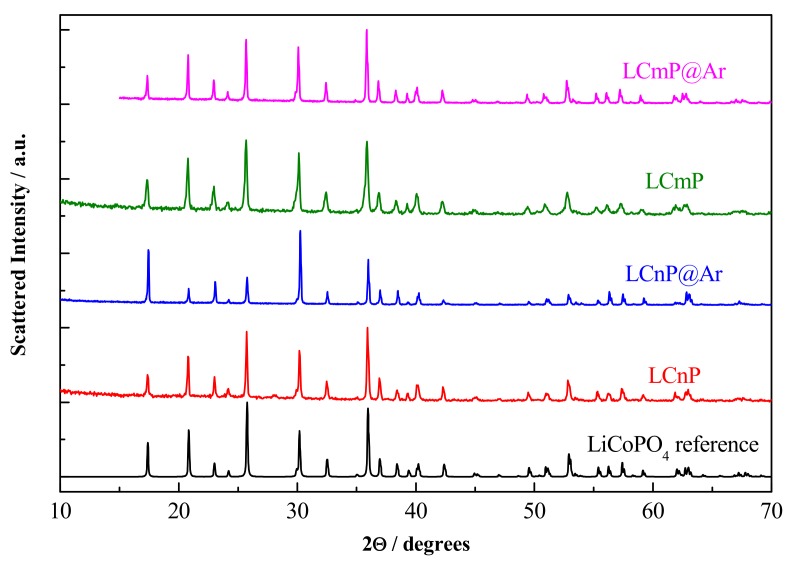
Patterns of the substituted samples before and after annealing under Ar. LiCoPO_4_ pattern is also reported as a reference.

The X-Ray Diffraction patterns measured on the samples substituted with Mn and Ni (LCmP and LCnP) and on the samples substituted and annealed under Argon flow (LCmP@Ar and LCnP@Ar) are reported in Figure 2.

The comparison with the XRD pattern of LiCoPO_4_ shows that all reflections in all patterns can be indexed by a single olivine phase with a *Pnma* space group. The lack of unindexed peaks confirms the phase purity of all the samples and the absence of contaminants above 2–3 vol.%, which is the typical detection limit for phase identification by powder XRD [21]. A detailed structural analysis of all the synthesized materials has been carried out by Rietveld refinement of these diffraction data, assuming as a starting point the stoichiometries obtained from the experimental data which are reported in Table 1.

The results of Rietveld refinement for the four materials are reported in Table 2 compared with the reference undoped LCP one. The fittings are shown in the Supplementary Material (Figures S1–S4). The cell volume values obtained for the two substituted samples are well comparable within the errors, as well as the cell parameters obtained for the *a* and *b* axes, while the Ni substitution induces a slight increase (around 0.1%) on the *c* axis compared to the Mn substituted sample. Both substitutions, however, induce an expansion of the cell volume compared to the undoped LCP sample, whose cell volume has been previously reported to be 284.3 ± 0.4 Å^3^. Moreover, Ni or Mn substitution also induce an isotropic expansion along the three axes: 0.1% along the *a* axis, 0.15% and 0.17% along the *b* and the *c* axis, respectively. This confirms that substitution affects the long-range ordering of the lattice. It can be noticed that the substitution effects on the cell parameters and volumes are very similar. Nevertheless, the size of the two substitution elements is either bigger or smaller than the substituting cobalt. The samples treated in argon, instead, display different behavior. In particular, the sample LCmP@Ar shows an isotropic expansion of the cell volume of about 0.5% compared to the non-treated LCmP sample. On the contrary, the LCnP@Ar sample presents a contraction of the cell volume compared to the starting non treated LCnP sample. This contraction, about 0.4%, seems to be slightly anisotropic since the *a* and *b* parameters decrease of about 0.15% (0.17%), whereas the *c* cell parameter is unaltered.

A similar anisotropic contraction induced by annealing in Argon has been reported for Fe-substituted LCP [14]. The cell shrinking in Fe-substituted LCP is coupled to a decrease of the anti-site cationic disorder, similar to what observed in Ni-substituted samples. This behavior could be explained by the very close values of the Shannon radii of nickel and iron atoms (0.645 Å for Fe^3+^ and 0.690 Å for Ni^2+^), both lower than the Shannon radius of the Co^2+^, which is 0.745 Å. Conversely, the manganese presents a larger radius value, around 0.830 Å for Mn^2+^. The structural results from the Rietveld refinements of the XRD data allow the calculation of the bond distances which have been reported in Table 3. Despite the very small differences among values obtained for the samples before and after annealing, one may notice that the thermal treatment induces a slight increase of the metal-oxygen bond distances of the Mn substituted LCP and a decrease of these distances when the LCP is substituted with Ni. However, it must be noted that the estimated M-O bond distances are unavoidably weighted means that include both the Co-O and the Mn(Ni)-O first coordination shells. Indeed, the XRD technique is unable to finely decouple the possible alteration around metal ions in the olivine lattice, but the X-Ray absorption analysis can provide more details about the local structure around the transition metal ions.

### 2.2. XANES

**Figure 3:**
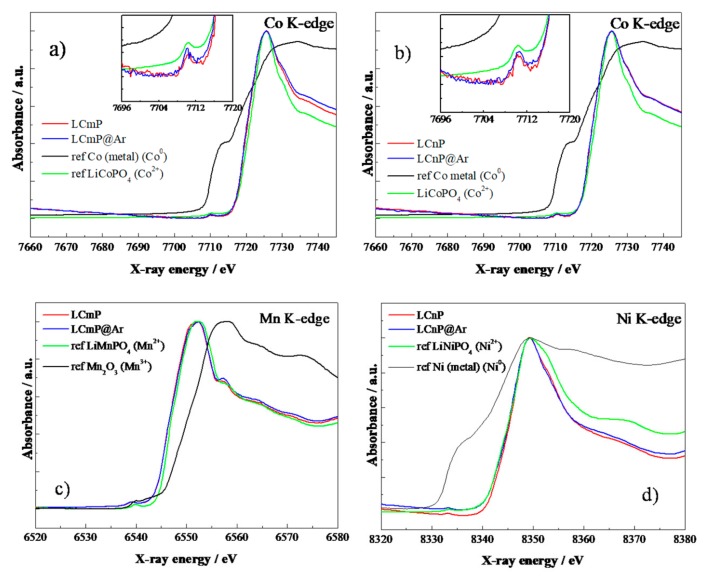
XANES spectra of (**a**) LCmP and LCmP@Ar at the Co K-edge, (**b**)LCnP and LCnP@Ar at the Co K-edge, (**c**) LCmP and LCmP@Ar at the Mn K-edge, (**d**)LCnP and LCnP@Ar at the Ni K-edge. In the insets, the pre-edge regions are shown.

The Co K-edge and Mn(Ni) K-edge absorption data are presented in Figure 3.

For each spectrum, the intensity of the main absorption peak has been normalized to the peak maximum and samples compared using E_0_, defined as the energy at half the height of this absorption peak [14]. The standards used for energy calibration contained Co^0^, Co^2+^, Mn^2+^, Mn^3+^, Ni^0^, Ni^2+^.

In Figure 3, all the Co edges reveal the Co 1s→4p white lines that are apparently unaltered regardless of the eventual substitution or annealing at high temperature.

The dipole forbidden pre-edge peaks, attributable to the Co 1s→3d transition, follow an identical trend compared to the white line, thus indicating the retention of the Co^2+^ state seen in the calibration standard [22]. Moreover, Figure 3c,d show a similar behavior also for the lines measured both at the Mn and Ni K-edge, with both pre-edge and E_0_ values indicating the +2 oxidation state for both the substitutional metal atoms. This oxidation state is retained even after the annealing under argon. This behavior is different from what previously reported for Fe substituted LCP, where the iron oxidation shifted to 3+ after similar annealing [14]. However, the oxidation of iron is expected to occur in the experimental conditions used in the annealing procedure, [3] whereas Mn^2+^ and Ni^2+^ are more stable and less prone to oxidize [23].

### 2.3. EXAFS

**Figure 4:**
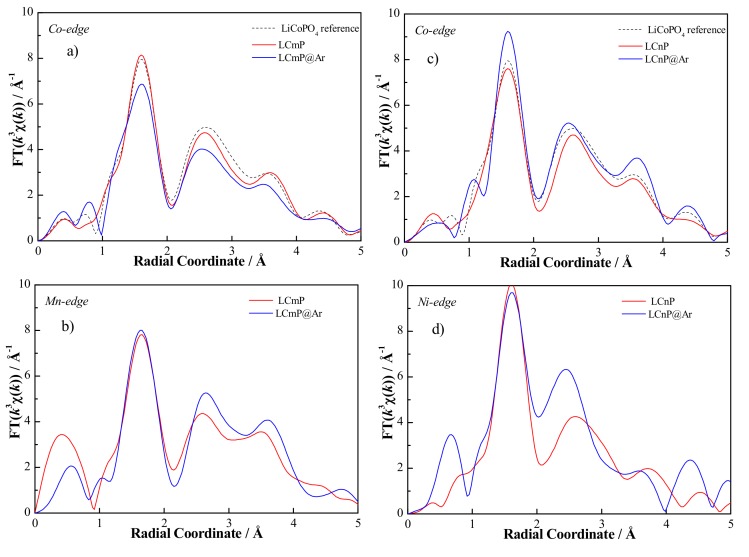
Radial distribution functions obtained after the Fourier transformation of *k*^3^*χ*(*k)*: (**a**) Co-edge and (**b**) Mn-edge of LCmP and LCmP@Ar samples; (**c**) Co-edge and (**d**) Ni-edge of the LCnP and LCnP@Ar samples. In the case of the Co edges a reference radial distribution function is shown corresponding to the undoped LiCoPO_4_ lattice [14].

The obtained radial distribution functions for the four samples are shown in Figure 4 for the Co, Mn, and Ni-edges.

All Co, Mn, and Ni-edges show a strong peak between 1 and 2 Å, and three further peaks at larger distances, in the range between 2 and 5 Å, with decreasing intensity. All the plotted radial structures present similar shape, suggesting a similar local structure around both scattering atoms, i.e., Mn, Ni, and Co, in all the four samples. Limited changes are observed in both LCmP@Ar and LCnP@Ar compared to the pristine material before annealing in Ar. After annealing a slight shift to lower distances of the peaks centered at 2.5 and 3.5 Å in Co-edges for both Mn- and Ni-substituted materials is observed. On the contrary, the trend observed in the Mn- and Ni-edges is opposite: in both cases, a shift in the position of the same two peaks centered at 2.5 and 3.5 Å is observed respectively to larger and smaller distances for Mn and Ni substituted samples.

**Figure 5:**
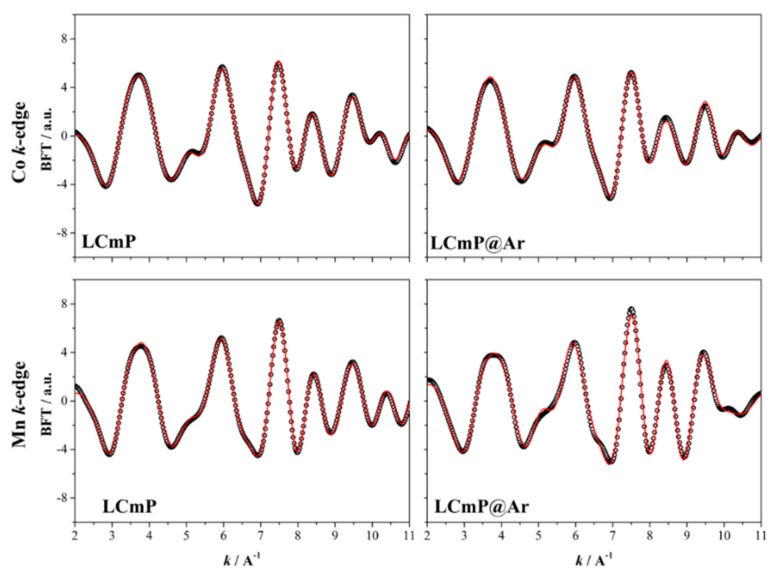
Points and best-fit line for the BFT of the k^3^-weighted Co and Mn K-edge signal for samples LCmP and LCmP@Ar.

**Figure 6:**
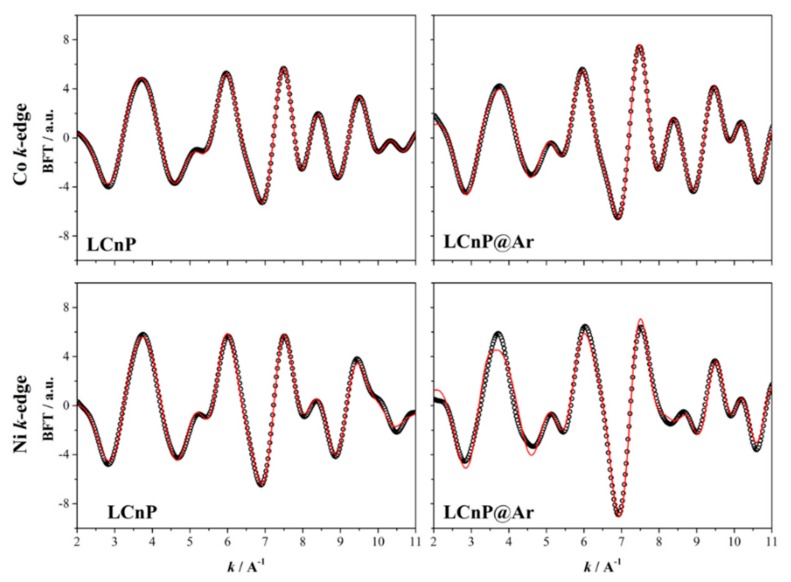
Points and best-fit line for the BFT of the k^3^-weighted Co and Ni K-edge signal for samples LCnP and LCnP@Ar.

The present data have been analyzed adopting a model already used by us for LCP and iron substituted LCP [14]. This model takes into account a first neighbor’s shell constituted by six oxygen atoms, located at three different distances. A second neighbors shell modeled by five phosphorous located at two different distances and six oxygen atoms located at a slightly higher distance and a third shell due to six metal atoms (Co/Ni/Mn), four at a shorter distance (~3.5 Å) from the scattering center and two at longer distance (~4.7 Å). This model is closer to the crystallographic structure than all the other models previously implemented to describe scattering paths around metal atoms in olivine based materials [14,24,25] and in particular it does not neglect a significant contribution due to scattering from the six oxygen second-neighbors thus providing a better description of the EXAFS signals [14]. The EXAFS fitting parameters for all the shells were the bond distances and the mean square relative displacements, whereas the coordination numbers were fixed. Figure 5 and Figure 6 show, for LCmP and LCnP respectively, the best fit results from quantitative analyses performed on the curves of the inverse Fourier transformation in the k range according to the peaks appearing in the radial structure.

The fitted distances between the scattering atoms are summarized in Table 4 for the LCmP based samples and in Table 5 for the LCnP based samples. The fitted thermal factors (Debye-Waller coefficients) are summarized in the Supplementary Material.

In line with the findings from EXAFS and XRD on LCP, iron substituted LCP and LiFePO_4_ [14,25], the metal-oxygen distances obtained for the first shell are slightly lower than the metal-oxygen distances calculated from the Rietveld refinements (respectively ranging in 1.84–2.19 vs. 2.05–2.23 Å).

At the Co K-edge, the bond distances in the first Co-O coordination shell are very close for the two substituted samples, and they are both slightly lower than the Co-O distances obtained by means of the same model for the undoped LCP [14]. The annealed samples instead show some differences when substituted with different elements. Indeed, the annealed LCmP@Ar shows a small isotropic increase of these distances, accompanied by a decrease of the distortion of the CoO_6_ octahedra being the three fitted distances scattered in a smaller interval, i.e., 1.96–2.18 vs. 1.90–2.16 Å before annealing. On the contrary, the Ni substituted sample displays an increase of the distortion of the CoO_6_ octahedra, being the three fitted distances scattered in a larger interval, i.e., 1.84–2.17 vs. 1.91–2.16 Å for LCnP@Ar and LCnP samples, respectively.

At the Mn K-edge, the first shell Mn-O bond distances increase, compared to the metal-oxygen distances obtained at the Co K-edge but slightly decrease after annealing. Turning to the Ni-substituted samples, instead, at the Ni K-edge, the Ni-O distances slightly decrease, as already observed for Fe substitution but increase after annealing. The annealing also decreases the octahedral distortion around the nickel-centers, being Ni-O distances scattered in a narrower interval (1.97–2.14 Å) for the LCnP@Ar sample than for the starting LCnP sample (1.86–2.15 Å).

Overall the TMO_6_ octahedra are very similar in all the four samples: after annealing, it is remarkable the reduction of their distortions with the exception of the case of the CoO_6_ ones in the LCnP@Ar sample. Moreover, one may interesting consider that after annealing the mean Co-O distances are 2.07 and 2.01 Å in the LCmP@Ar and LCnP@Ar samples, respectively whereas the Mn-O and Ni-O mean distances are in both cases 2.05 Å.

Turning to the Co-P scattering, all distances obtained for all the four samples are very close within the errors and very close to the same values previously reported for pure LCP [14]. On the contrary, the Mn-P and Ni-P distances are respectively expanded and contracted compared to the Co-P ones, in particular in the samples annealed in Ar. Overall the annealing seems to shift the PO_4_ octahedra closer to the Ni^+2^ ions and far from Mn^2+^. Being the NiO_6_ and MnO_6_ octahedra very similar in size after the annealing (see above Table 4 and Table 5), the shift of the PO_4_ tetrahedra is likely induced by the different size of the surrounding CoO_6_ octahedra (i.e., larger for LCmP@Ar and smaller for LCnP@Ar). Two concurring effects may play a major role in the tuning of the modulation of bonds (both in terms of distances and angles) around the transition metal centers and the surrounding coordination shells: ion size and bond ionicity (i.e., more ionic TM-O bond typically show smaller distances). Therefore, these trends are driven by the balancing between the different Co^2+^, Mn^2+^ and Ni^2+^ sizes (0.745, 0.830 and 0.690 Å, respectively) and the increase of approximately 20% of the difference in the electronegativity of the Mn-O bond compared to the Co-O/Ni-O ones.

### 2.4. Galvanostatic Cycling

**Figure 7:**
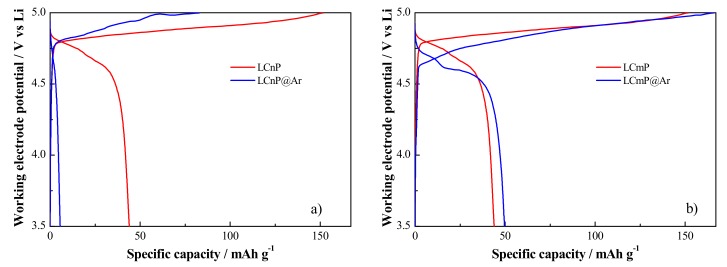
Cell voltage profile in galvanostatic conditions of the (**a**) Ni- substituted and (**b**) Mn-substituted samples before and after the annealing under Ar.

The results of the galvanostatic test performed at a 0.1 C rate (1C = 167 mA g^−1^) of lithium cells using the four samples are reported in Figure 7. The benchmark data for the undoped LCP material has been reported by us in [14] and it is here omitted to avoid redundancies.

The charge/discharge profiles of the Mn- and Ni-substituted samples before annealing show a partially reversible voltage pseudo-plateau centered around 4.75 V vs. Li, likely due to the redox couple Co^3+^/Co^2+^. Apparently, the redox activities of the Mn^2+^/Mn^3+^ or Ni^2+^/Ni^3+^ couples are not clearly identified. The Mn-redox activity is expected to be centered at about 4.1 V vs. Li whereas nickel at V > 4.9–5.0 V vs. Li. This evidence agrees with the findings reported for similar systems like the Fe-substituted LCP phase [14] where the Fe^2+^/Fe^3+^ redox couple does not give any clearly observable electrochemical fingerprint at 3.5–3.8 V vs. Li.

After annealing under Ar, both the substituted LCmP@Ar and LCnP@Ar phases show changes in the electrochemical activities. In particular, the Mn -substituted one shows a decrease below 4.6 V vs. Li of the mean redox potential and the appearance of multiple pseudo-plateau both in charge (at about 4.7, 4.75 and 4.85 V vs. Li) and discharge (at about 4.7 and 4.6 V vs. Li) [1,14]. The LCnP@Ar sample highlights a major deterioration of the electrochemical activity after the annealing with an increase of the oxidation potential and an almost irreversible reactivity after the charge (lithium de-insertion).

Turning to the supplied capacity, upon charge three out of four materials approach the theoretical capacity values (~167 mAhg^−1^) thus suggesting an almost complete lithium de-insertion apart the LCnP@Ar sample that barely reach a nominal stoichiometry of Li_0.4_Co_0.9_Ni_0.1_PO_4_ at the end of the charge step.

Unfortunately, in all cases, the reversibility of the redox reaction (Li-insertion) is poor and never exceeds 50 m∙Ah g^−1^, which is almost the half of the capacity of the undoped LCP material [14]. In the case of the Mn-substituted sample, the Ar treatment slightly improves both the charge and discharge capacities but reduces the overall reversibility in terms of coulombic efficiency. Remarkably the reversibility of the lithium de-insertion/insertion is completely lost in the Ni-substituted sample after the annealing.

## 3. Discussion

**Figure 8:**
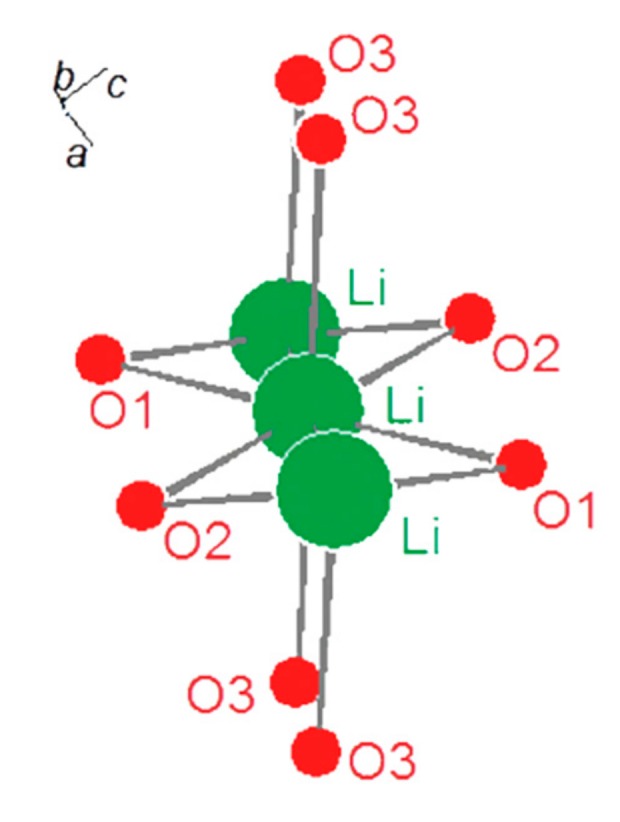
Ions diffusion path along the (010) lattice direction of the olivine phase. The shown O1, O2 and O3 oxygen atoms (4c/4c/8d lattice sites, respectively) are the closest first neighbors of the Li-centres (4a lattice site) and limit the size of the diffusion channel.

The electrochemical performance of the olivine materials is closely affected by subtle changes in the local ordering of the crystal structure and mainly by disorder and obstruction of the Li^+^ diffusion paths. In fact, lithium ions diffuse in the structure only through 1D channels along the (010) lattice direction, as shown in Figure 8. The size of these diffusion paths can be estimated by multiplying the O3-O3 axial distances by the O1-O2 equatorial distances (see below in the Materials and Methods section for clarifications concerning the oxygen atomic sites in the olivine lattice). Moreover one should also recall that the electronic disorder induced by partial TM substitutions in the lattice may alter the electronic properties at the Fermi level, thus affecting the electronic conduction properties.

In a previous publication, we demonstrated the beneficial effects of the Fe^3+^ aliovalent substitution on the electrochemical performance of the LiCoPO_4_ olivine phase. Being the local coordination and the long-range lattice structure, both altered by substitution as well as the point defect concentrations, we demonstrated how the balancing among the concurring effect may hinder or boost the ability of the final material to reversibly exchange lithium in cells. Here both the Mn^2+^ and Ni^2+^ isovalent substitution negatively affects performance and therefore our structural analysis can rationalize the origin of this failure.

Structural features that may impact the lithium diffusion through narrowing of the (101) channels or their obstruction are:

(a) The cell contraction along the *a* and *c* lattice axes,
(b) The expansion/deformation of the TMO_6_ octahedra that share the O_1_ and O_3_ centers with the LiO_6_ octahedra,
(c) The shift or deformation of the PO_4_ tetrahedra that share the O_2_ centers with the LiO_6_ octahedra,
(d) The occurrence of extended anti-site disorder between the 4a and the 4c sites respectively occupied by the Li and TM ions,
(e) The occurrence of lithium vacancies on the 4a lattice site.

Effects (a-b-c) are closely interrelated and can be strongly affected also by the possible modulation of the local ordering induced by extensive metal substitution. Point defects like anti-sites (effect d) strongly limit diffusion and are generally recognized as one of the most relevant effects that damage the reversibility of the electrochemical lithium de-insertion/insertion. On the contrary vacancies on the lithium sublattice (effect e) facilitates diffusion by increasing the number of voids that can support the metal hopping. Our XRD structural refinements allow estimating the alteration of the size of the Li^+^ diffusion channels that can be estimated by the area of the planar projection of the O_2_-Li-O_3_ and O_3_-Li-O_3_ distances perpendicular to the (010) lattice direction. In the case of the undoped LCP lattice the size of the diffusion channel is about 20.3 Å^2^: the iso-valent substitution with Mn or Ni narrows its size of about 4–5% to 19.3 and 19.6 Å^2^ for the LCmP and LCnP samples, respectively. After annealing, the size of the (010) diffusion channels increases to 20.4 and 19.9 Å^2^ for the LCmP@Ar and LCnP@Ar samples, respectively.

EXAFS fittings suggest that the size of the CoO_6_, MnO_6,_ and NiO_6_ octahedra are very similar and only marginally affected by annealing. On the contrary, compared to the benchmark 3.16 Å mean distance between Co-P in LCP, all the Mn and Ni substituted samples show larger TM-P mean distances, thus suggesting an outer shift of the PO_4_ tetrahedra likely narrowing the Li^+^ diffusion channels.

Turning to point defects concentration estimated by the Li content obtained by the ICP composition analyses (see Section 4) or Rietveld refinements (anti-site disorder), the LCmP and LCmP@Ar samples show an increase in the Li^+^ vacancies whereas LCnP and LCnP@Ar compositions suggest negligible voids in the 4a lattice site. On the contrary, anti-site defects (See Table 2) are in all the four cases strongly increased compared to both the undoped LCP and the optimal Fe^3+^substituted one.

Overall in the case of the Mn-substitution in the olivine lattice, the lithium diffusion channels shrink, the PO_4_ tetrahedra shift towards the Li^+^ ions and the anti-site disorder increases. These negative effects are only partially counterbalanced by the increase in the vacancies on the 4a atomic site (see Section 4). Passing from Mn to Ni substitution this detrimental combination of structural features is worsened as the composition of the samples suggests the absence of any relevant voids in the lithium sublattice.

## 4. Materials and Methods

### 4.1. Synthesis Route

LCP based materials have been synthesized by a solvothermal route already implemented by the present authors to synthesized undoped and Fe substituted LCP [14,26,27]. Two water solutions, one containing LiOH•H_2_O (solution A) and another containing LiH_2_PO4 and CoSO_4_•7H_2_O (solution B) have been added in sequence to ethylene glycol (EG) under vigorous stirring (EG:H_2_O *v/v* ratio = 2:1, final volume 30 mL). The molar ratios of LiH_2_PO_4_, CoSO_4_•7H_2_O and LiOH•H_2_O have been optimized to the values 1:1:1.75. The Co^2+^ concentration in the final EG:H_2_O solution is 0.1 M. The obtained purple suspension has been sealed into a 45 mL Teflon-lined autoclave and heated in an oven at 220 °C for 15 h. The products have been filtered, washed with H_2_O/ethanol and dried in the oven. Mn(Ni) substituted materials have been obtained by using as additional reagent MnSO_4_/(NiSO_4_)•7H_2_O and by setting the relative molar ratio of CoSO_4_ and MnSO_4_/(NiSO_4_) to 0.9:0.1. The rationale of this synthesis route, adapted from the original version from Grey and co-workers [28], is to precipitate the olivine phase at low temperature in a highly crystalline form using a slightly acidic lithium-rich solution with moderate dielectric constant. Possible lithium over/under-stoichiometry may occur due to the complex interplay between thermodynamic and kinetic driving forces, eventually moderated by the possible tendency to form defects induced by substituents. The composition of the synthesized material has been determined by inductively coupled plasma−atomic emission spectroscopy (ICP-AES) using a Vista MPX Rad−VARIAN instrument following an experimental protocol described elsewhere [3,14] and adopting proper evaluations to take into account possible minor alteration of the stoichiometries for samples annealed at high temperature compared to the pristine ones [3,14]. Both Mn or Nisubstituted LCP samples have been also annealed at high temperatures under Air-flow (80 mL/min) at 700 °C for 1 h.

Four samples have been synthesized and studied: a summary of the synthesis conditions, estimated stoichiometries from ICP-AES and the corresponding sample codes adopted throughout the text are presented in Table 1 (see previous sections).

### 4.2. X-ray Diffraction Experiments

XRD experiments have been carried out by using a laboratory diffractometer Phillips X-pert Plus using CuKα radiation. XRD has been recorded in the 10–70° 2θ range with steps of 0.015° (time/step of 6 s). Structure refinement has been performed by the Rietveld method using GSAS [29] starting from the olivine lattices of the prototypal LiCoPO_4_ phase (orthorhombic unit cell with space group no. 62 Pnma, Li atoms in 4a (0, 0, 0), Co in 4c (x_TM_, ¼, z_TM_), P in 4c (x_P_, ¼, z_P_) and three oxygen atoms in O1 in 4c (x_O1_, ¼, z_O1_) O2 in 4c (x_O2_, ¼, z_O2_), and O3 8d (x_O3_, y_O03_, z_O3_)).

In the Rietveld refinements, few constraints have been adopted: in particular, the Li:TM:P ratios have been fixed to the estimated stoichiometries (see Table 1), and thus occupancies have not been freely optimized: only the anti-site Li/Co cationic disorder has been refined. Debye-Waller factors have been optimized by applying two constraints: all cations (i.e., Li, Co, Mn, Ni) have been constrained to the same values as well as all atoms (i.e., P and O) constituting the PO_4_ tetrahedra. In summary, the following parameters have been optimized: (a,b,c) lattice parameters, Caglioti coefficients, Gaussian-to-Laurentian coefficients, Debye-Waller factors for cations and for PO_4_tetrahedra, the Li/Co cationic disorder and the atomic positions.

### 4.3. X-Ray Absorption Experiments

X-ray absorption spectroscopy experiments were carried out at the Italian beam Line for X-ray absorption spectroscopy (LISA) of the European Synchrotron Radiation Facility (ESRF, Grenoble). All samples were mixed with cellulose and pressed to obtain pellets, which were measured at room temperature in transmission mode at the Co K-edge (7712 eV). Manganese and nickel substituted samples were also measured in fluorescence mode respectively at the Mn K-edge (6539 eV) and Ni K-edge (8340 eV). The same sample was used for spectra at both absorption edges in order to rule out spurious contributions. Energy calibrations have been carried out using Co and Ni and Mn metal foils as a reference. Standard commercial Mn_2_O_3_, LiMnPO_4_ and LiNiPO_4_ samples were also measured as references for XANES analysis.

The analysis of the XAS signals has been performed by the Viper program [30]. The absorption spectrum below the pre-edge region has been fitted to a straight line while the background contribution above the post-edge region has been fitted to a polynomial function. After subtraction of the fitted background, the absorption spectra have been normalized and transformed in the k space χ(k). The k^3^ weighted EXAFS oscillations, k^3^χ(k), were Fourier transformed (FT) in the k range between 1.72 and 11.87 Å^−1^ for the Co edge, between 2.03 and 10.25 Å^−1^ for the Mn edge and between and 87 Å^−1^ for the Ni edge using a Hanning window function to obtain the magnitude plots of the EXAFS spectra in R-space (Å). The analysis has been restricted to the first four FT peaks by inverse Fourier transforming (Fourier filtering) the data in the R range between 0.8and 4.85 Å (0.98 and 4.87 Å for the Mn edge, 0.98 and 4.87 Å for the Ni edge). The fit in the k-space has been performed using standard single-scattering EXAFS formula with amplitude and phase functions generated from ab initio code FEFF integrated into ARTEMIS (IFEFFIT package) [31].

Data have been fitted assuming eight scattering paths according to a model already successfully used for LCP and Ni-substituted LCP [14]. This model adopts a first neighbors shell constituted by six oxygen atoms, located at three different distances; a second neighbors shell modeled by five phosphorous located at two different distances and six oxygen atoms located at a slightly higher distance and a third shell due to metal atoms (Co/Ni/Mn). In particular, for this last shell, we assumed four metal atoms at a shorter distance (~3.9 Å) from the scattering center and two at a longer distance (~4.6 Å). For all the considered shells, the fitting parameters are the bond distances and the mean square relative displacements whereas the coordination numbers are fixed. The interatomic distances (D) used as starting points for the fit are the values obtained by the diffraction experiments.

### 4.4. Electrochemical Tests

The electrode films have been deposited on an aluminum foil by doctor-blading a slurry composed of 80% of the active material (AM), 10% of PVdF-HFP (Kynar Flex 2801, Arkema, Colombes, France) and 10% of Super P carbon in tetrahydrofuran (THF, Sigma-Aldrich, St. Luis, MO, USA). Percentages are wt%. The mass loading over the aluminum foil is approximately 2–3 mg cm^−2^. All the samples have been tested in galvanostatic cycling in lithium cells in the cell potential range 3.5–5 V at 0.1 C rate (1C = 167 mA g^−1^). The galvanostatic cycling experiments have been carried out with an MTI galvanostat using ECC-STD flat cells (EL-CELL Gmbh, Hambourg, Germany). The cells have been prepared in an Iteco Engineering Ar-filled glovebox, by coupling the electrode under test with a lithium foil counter electrode in 1 M LiPF_6_ ethylene carbonate/dimethyl carbonate (EC:DMC) electrolyte solution (Solvionic), soaked on a Whatman^TM^ glass fiber separator (Whatman, Maidstone, Great Britain).

## 5. Conclusions

In this study, we tackled the analysis of the structure to function relation in iso-valent substituted LiCoPO_4_olivines. Crystalline olivine phases derived by the LiCoPO_4_ stoichiometry by substituting 10% of the cobalt ions with Mn^2+^ or Ni^2+^ have been synthesized and tested in lithium cells as positive electrode materials. Furthermore, a detailed structural analysis by XRD, XANES and EXAFS has been carried out in order to investigate the alteration of the main structural features induced by substitution. XRD analysis suggests that substitution induces an expansion of the lattices and an increase of the antisite disorder between lithium and transition metal ions in the structure. XAS data highlight negligible electronic disorder but a relevant modulation in the local coordination around the different metal ions. These results are discussed suggesting that iso-valent substitution leads to a detrimental combination of structural changes: (a) the narrowing of the lithium diffusion channels, (b) the increase in point defects and (c) the appearance of local structural deformations. All these effects induced a dramatic loss in performance in cells in contrast to the optimal balancing observed by us previously in the case of the aliovalent Fe^3+^ substitution.

## Figures and Tables

**Table 1:** Coding and estimated stoichiometries.

Sample Coding	T_ann_ Under Ar Flow	Li:Co:Mn:Ni Ratio by ICP-OES	CoSO_4_:Mn(Ni)SO_4_ Molar Ratio	Experimental Stoichiometry
**LCmP**		0.933:1.001:0.104:0	9.0:1.0	Li_0.89_(Co_0.9_Mn_0.1_)_1.04_PO_4_
**LCmP@Ar**	700 °C	0.936:1.002:0.105:0	9.2:1.0	Li_0.89_(Co_0.9_Mn_0.1_)_1.04_PO_4_
**LCnP**		1.055:0.933:0:0.096	9.0:1.0	Li_1.02_(Co_0.9_Ni_0.1_)_0.99_PO_4_
**LCnP@Ar**	700 °C	1.051:0.941:0:0.099	8.9:1.0	Li_1.01_(Co_0.9_Ni_0.1_)_1.00_PO_4_

**Table 2:** Rietveld Refinement results for all samples. Errors on the last digit of the cell volume are reported in parenthesis (Occ. stands for occupancy factor, vac stands for unoccupied/vacant sites).

Stoichiometry and Vacancy Occupation Factor on the (4a) Wycoff Site	Cell Volume Å^3^	Cell Parameters Å	Antisite Disorder Occupancy (4a/4c)	_w_R_p_
**Sample LCmP**				
Li_0.89_ (Co_0.9_Mn_0.1_)_1.04_PO_4_ Occ.(vac, 4a) = 0.05	286.1 (2)	a = 10.232 (1) b = 5.935 (1) c = 4.710 (1)	0.046	0.0489
**Sample LCmP@Ar**				
Li_0.89_(Co_0.9_Mn_0.1_)_1.04_PO_4_ Occ.(vac, 4a) = 0.05	287.6 (1)	a = 10.250 (1) b = 5.951 (1) c = 4.716(1)	0.048	0.0422
**Sample LCnP**				
Li_1.01_(Co_0.9_Ni_0.1_)_0.99_PO_4_ Occ.(vac, 4a) = 0	286.2 (1)	a = 10.230 (1) b = 5.934 (1) c = 4.715 (1)	0.06	0.0503
**Sample LCnP@Ar**				
Li_1.01_(Co_0.9_Ni_0.1_)_0.99_PO_4_ Occ.(vac, 4a) = 0	285.2 (1)	a = 10.212 (1) b = 5.925(5) c = 4.713 (4)	0.045	0.0495
**Reference LCP** [14]				
LiCoPO_4_	284.3 (4)	a = 10.207 (3) b = 5.923 (1) c = 4.702 (3)	0.006	0.0250

**Table 3:** Distances (Å, estimated mean uncertainty ±0.01Å) and degeneracies (in parentheses) calculated from structural refinements of the XRD data.

LCmP	LCmP@Ar	LCnP	LCnP@Ar	LCP [14]
**Mn-O**				
2.08 (x2)	2.08 (x2)	2.11 (x2)	2.13 (x2)	2.06 (x2)
2.08	2.05	2.05	2.05	2.09
2.08	2.14	2.21	2.16	2.17
2.21 (x2)	2.21 (x2)	2.23 (x2)	2.18 (x2)	2.20 (x2)
**Mean**				
2.12	2.13	2.16	2.14	2.13
**M-P**				
2.77	2.79	2.78	2.76	2.79
**M-Li**				
3.22	3.22	3.22	3.20	3.21

**Table 4:** Between scattering atoms in Å obtained by best fit for LCmP based samples. CN is the coordination number, M-Z represents the central absorber (M, that is Co or Mn respectively in the Co K-edge and the Mn K-edge fits) and the scattering atom (Z, that is O, P/O or Co/Mn in the three shells considered). Statistical errors on distances are in all cases smaller than 0.01 Å.

Shell	M-Z	C-N	Co K-edge		Mn K-edge	
			LCmP	LCmP@Ar	LCmP	LCmP@Ar
	M-O	2	1.90	1.96	1.94	1.93
**1st shell**	M-O	2	2.01	2.06	2.05	2.04
	M-O	2	2.16	2.18	2.22	2.19
	M-P	1	2.81	2.82	2.83	2.90
**2nd shell**	M-P	4	3.26	3.29	3.27	3.28
	M-O	6	3.57	3.39	3.63	3.59
	M-M’	4	3.88	3.84	3.80	3.81
**3rd shell**	M-M’	2	4.76	4.74	4.63	4.42
**R factor (%)**			4.9	7.9	4.8	9.7

**Table 5:** Between scattering atoms in Å obtained by best fit for LCnP based samples. C-N is the coordination number. M-Z represents the central absorber (M, that is Co or Ni respectively in the Co K-edge and the Ni K-edge fits) and the scattering atom (Z, that is O, P/O or Co/Ni in the three shells considered). Statistical errors on distances are in all cases smaller than 0.01 Å.

Shell	M-Z	C-N	Co K-edge		Ni K-edge	
			LCnP	LCnP@Ar	LCnP	LCnP@Ar
	M-O	2	1.91	1.84	1.86	1.97
**1st shell**	M-O	2	2.01	2.01	2.00	2.05
	M-O	2	2.16	2.17	2.15	2.14
	M-P	1	2.86	2.84	2.84	2.77
**2nd shell**	M-P	4	3.27	3.27	3.29	3.20
	M-O	6	3.53	3.56	3.34	3.63
	M-M’	4	3.84	3.87	3.97	3.80
**3rd shell**	M-M’	2	4.71	4.75	4.86	4.72
**R factor (%)**			3.9	5.9	8.6	16.8

## Supplementary Materials

The following are available online, Figure S1. Rietveld refinement plot obtained for the LCmP sample. Figure S2. Rietveld refinement plot obtained for the LCmP@Ar sample. Figure S3. Rietveld refinement plot obtained for the LCnP sample. Figure S4. Rietveld refinement plot obtained for the LCnP@Ar sample. Table S1. Debye Waller factors fitted form the LCmP and LCmP@Ar samples on the Co and Mn-edges from XAS data. Table S2. Debye Waller factors fitted form the LCnP and LCnP@Ar samples on the Co and Ni-edges from XAS data.
