# Supplementary material for: Effect of Transitional Metals (Mn and Ni) Substitution in LiCoPO4 Olivines
Source: Molecules. 2020 Jan 30;25(3):601. doi: 10.3390/molecules25030601 (PMC7037934; doi:10.3390/molecules25030601)
Supplement: Supplementary file 1 [file molecules-25-00601-s001.pdf]

molecules

Supplementary Materials

Effect of transitional metals (Mn and Ni) substitution in$LiCoPO_4$olivines

Oriele Palumbo[1], Jessica Manzi[1], Daniele Meggiolaro,[2] Francesco M. Vitucci[1],Francesco Trequattrini[1,3], Mariangela Curcio[4], Annalisa Paolone[1]andSergio Brutti[5,*]

1 CNR-ISC, U.O.S. La Sapienza, Piazzale A. Moro 5, 00185 Rome, Italy; oriele.palumbo@roma1.infn.it(O. P.), manzi.jess@gmail.com (J.M.), Francesco.m.vitucci@gmail.com (F.V.); annalisa.paolone@roma1.infn.it (A.P.)
2 Computational Laboratory for Hybrid/Organic Photovoltaics (CLHYO) Istituto CNR di Scienze e Tecnologie Chimiche "Giulio Natta"(CNR-SCITEC), Via Elce di Sotto 8, 06123 Perugia, Italy; daniele.meggiolaro@iit.it
3 Department of Physics, University of Rome ''La Sapienza'', Piazzale A. Moro 5, 00185 Rome, Italy; francesco.trequattrini@roma1.infn.it
4 Department of Sciences, University of Basilicata, V.le dell'Ateneo Lucano 10, 85100 Potenza, Italy.; mariangela.curcio@unibas.it (M.C.)
5 Department of Chemistry, University of Rome ''La Sapienza'', Piazzale Aldo Moro 5, 00185 Rome, Italy.; sergio.brutti@uniroma1.it (S.B.)
* sergio.brutti@uniroma1.it; Tel +39-06-4991-3957

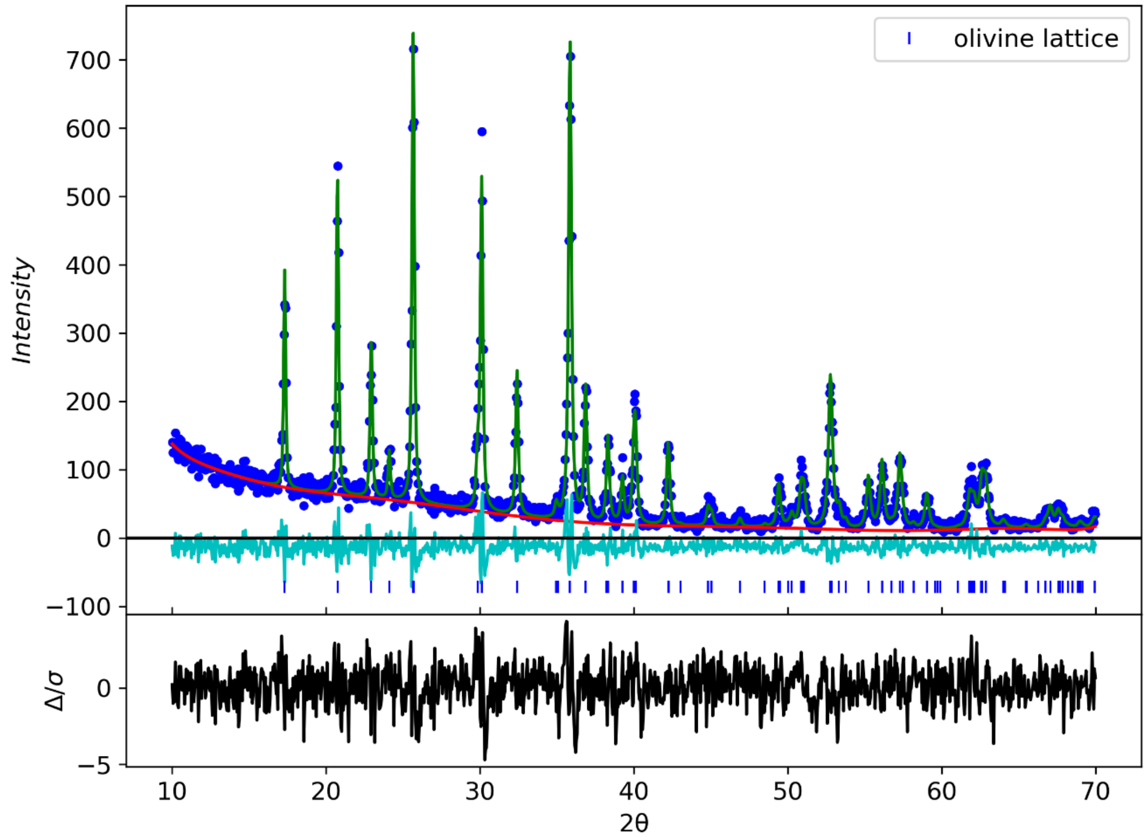

Figure S1. Rietveld refinement plot obtained for the LCmP samples.

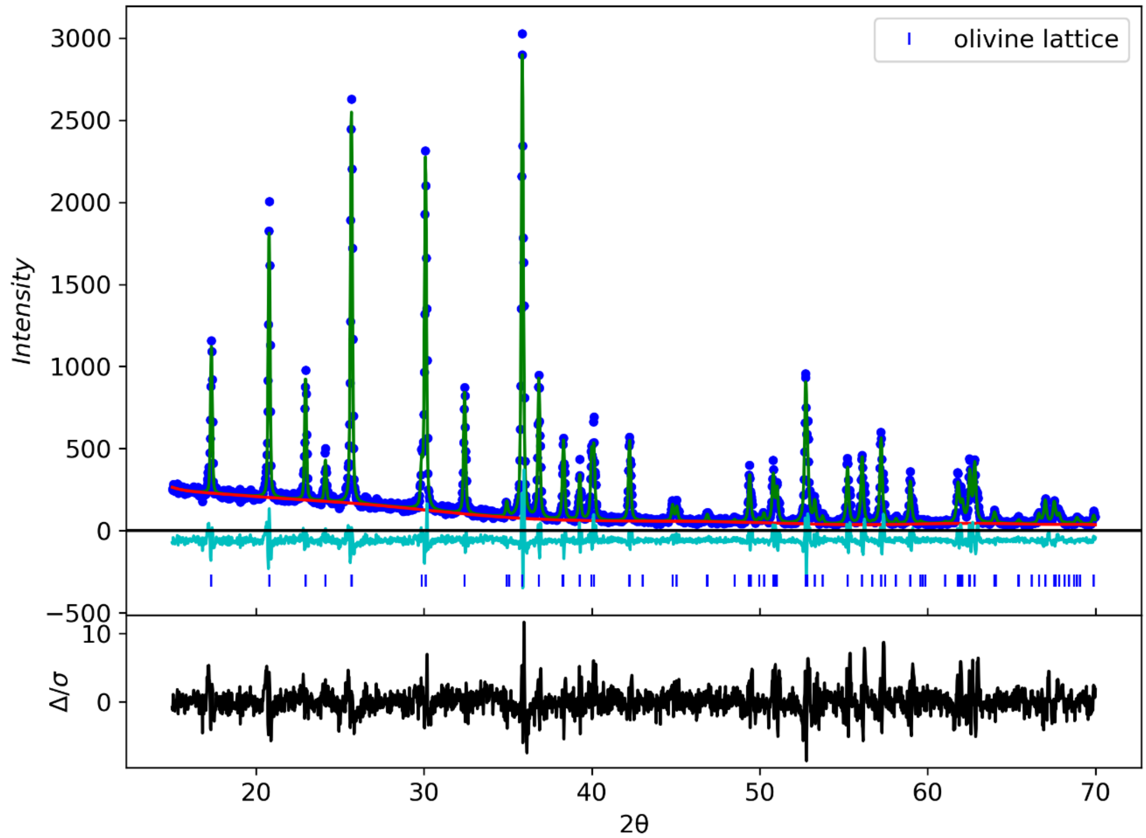

Figure S2. Rietveld refinement plot obtained for the LCmP@Ar samples.

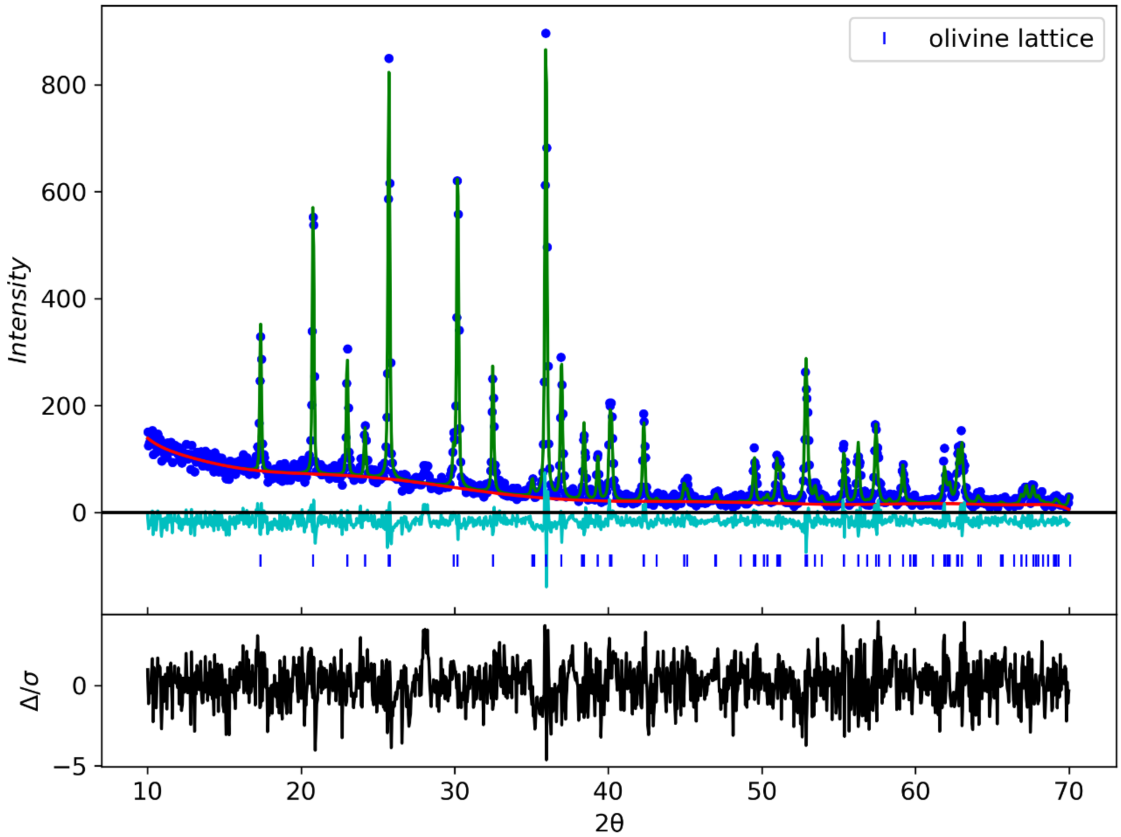

Figure S3. Rietveld refinement plot obtained for the LCnP samples.

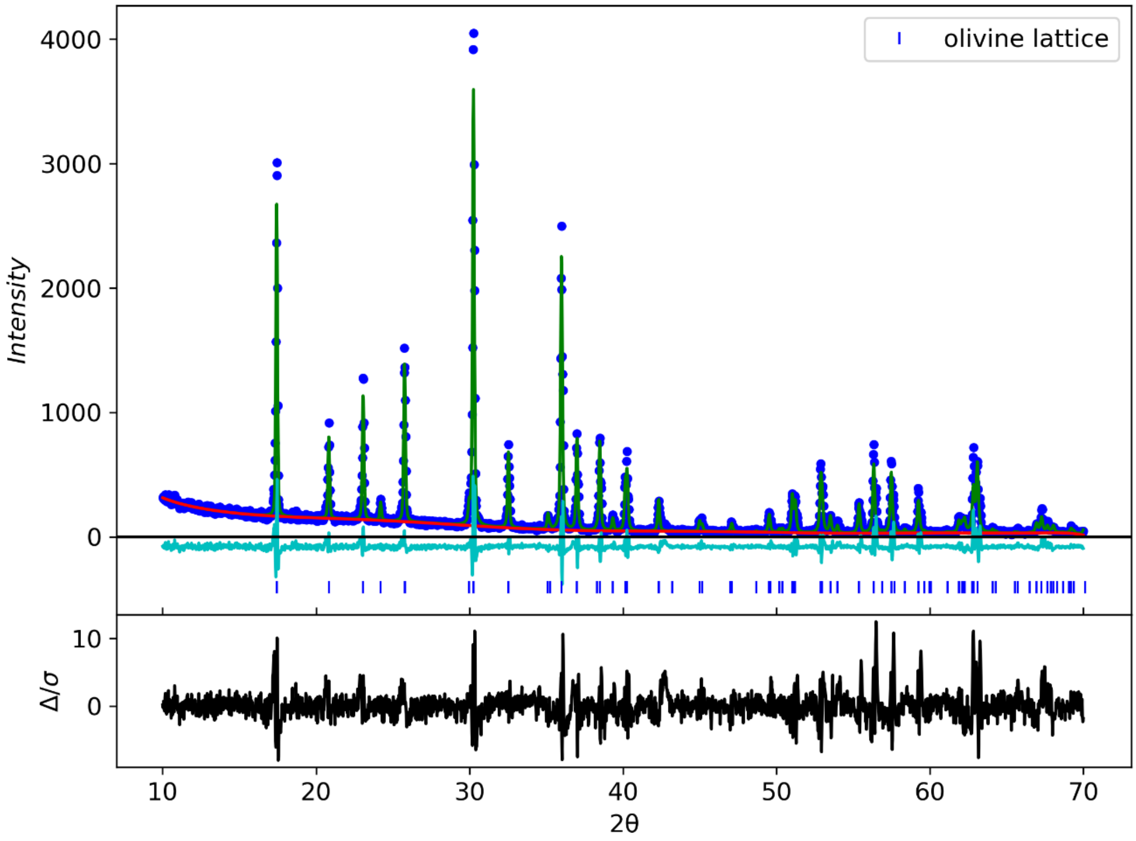

Figure S4. Rietveld refinement plot obtained for the LCnP@Ar samples.

Table S1. Mean square relative displacements in (σ^2 / 10^{-3}Å^2) obtained by best fit for LCmP based samples. CN is the coordination number; M-Z represent the central absorber (M, that is Co or Mn respectively in the Co K-edge and the Mn K-edge fits) and the scattering atom (Z, that is O, P/O or Co/Mn in the three shells considered). Statistical errors on distances are in all cases smaller than 0.2x10^{-3}Å^2

			**Co K-edge**		**Mn K-edge**	
**Shell**	**M-Z**	**C-N**	**LCmP**	**LCmP@Ar**	**LCmP**	**LCmP@Ar**
	M-O	2	8.9	3.2	7.7	2.8
**1st shell**	M-O	2	0.1	0.1	0.1	0.1
	M-O	2	1.6	2.1	1.7	0.1
	M-P	1	4.7	4.5	5.7	1.5
**2nd shell**	M-P	4	6.8	6.1	7.7	8.8
	M-O	6	35.7	20.6	27.9	14.5
	M-M′	4	10.6	14.1	8.4	8.5
**3rd shell**	M-M′	2	7.9	8.9	6.7	6.2
**R factor(%)**			4.9	7.9	4.8	9.7

Table S2. Mean square relative displacements in (σ^2 / 10^{-3}Å^2) obtained by best fit for LCnP based samples. CN is the coordination number; M-Z represent the central absorber (M, that is Co or Ni respectively in the Co K-edge and the Ni K-edge fits) and the scattering atom (Z, that is O, P/O or Co/Ni in the three shells considered). Statistical errors on distances are in all cases smaller than 0.2x10^{-3}Å^2

			**Co K-edge**		**Ni K-edge**	
**Shell**	**M-Z**	**C-N**	**LCnP**	**LCnP@Ar**	**LCnP**	**LCnP@Ar**
	M-O	2	8.1	18.8	14.8	1.2
**1st shell**	M-O	2	0.1	0.1	0.1	0.1
	M-O	2	0.4	2.0	0.4	0.1
	M-P	1	4.8	2.6	5.8	0.1
**2nd shell**	M-P	4	7.4	6.8	6.7	5.8
	M-O	6	28.7	22.4	27.6	22.8
	M-M′	4	11.4	8.4	14.9	13.0
**3rd shell**	M-M′	2	9.7	5.9	11.6	2.5
**R factor(%)**			3.9	5.9	8.6	16.8
